# (2*S*,4*R*,5*S*)-5-Allyl-4-hydroxy­tetra­hydro-2-furylmethyl *p*-toluene­sulfonate

**DOI:** 10.1107/S160053680902011X

**Published:** 2009-06-06

**Authors:** Evelyn Paz-Morales, Fernando Sartillo-Piscil, Angel Mendoza

**Affiliations:** a Facultad de Ciencias Químicas, Benemérita Universidad Autónoma de Puebla, Puebla, Pue., Mexico; b Centro de Química, ICUAP, Benemérita Universidad Autónoma de Puebla, Puebla, Pue., Mexico

## Abstract

In the title compound, C_15_H_20_O_5_S, the tetra­hydro­furan ring shows an envelope conformation. The crystal packing is stabilized by an inter­molecular O—H⋯O hydrogen bond, generating a ribbon structure along the *a* axis. Two weak inter­molecular C—H⋯O inter­actions are also observed.

## Related literature

For the synthesis of chiral tetra­hydro­furans bearing an allyl group at the C1 position, see: Romero *et al.* (2006 ▶); Sartillo-Melendez *et al.* (2006 ▶); Hernández-Garcia *et al.* (2009 ▶); Paz-Morales *et al.* (2009 ▶). For ring conformation analysis, see: Cremer & Pople (1975 ▶).
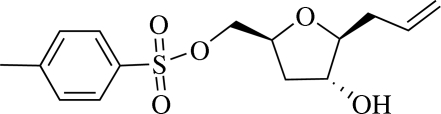


## Experimental

### 

#### Crystal data

C_15_H_20_O_5_S
*M* *_r_* = 312.37
Monoclinic, 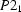

*a* = 5.9420 (12) Å
*b* = 16.966 (3) Å
*c* = 8.1980 (19) Å
β = 100.09 (2)°
*V* = 813.7 (3) Å^3^
*Z* = 2
Mo *K*α radiation
μ = 0.22 mm^−1^
*T* = 293 K
0.6 × 0.4 × 0.3 mm

#### Data collection

Bruker P4 diffractometer
Absorption correction: none
2127 measured reflections
1575 independent reflections
1099 reflections with *I* > 2σ(*I*)
*R*_int_ = 0.030
3 standard reflections every 97 reflections intensity decay: 7%

#### Refinement

*R*[*F* ^2^ > 2σ(*F* ^2^)] = 0.055
*wR*(*F* ^2^) = 0.126
*S* = 1.07
1575 reflections
194 parameters
1 restraint
H atoms treated by a mixture of independent and constrained refinement
Δρ_max_ = 0.26 e Å^−3^
Δρ_min_ = −0.23 e Å^−3^
Absolute structure: Flack (1983 ▶), 97 Friedel pairs
Flack parameter: −0.1 (2)

### 

Data collection: *XSCANS* (Siemens, 1994 ▶); cell refinement: *XSCANS*; data reduction: *XSCANS*; program(s) used to solve structure: *SIR2004* (Burla *et al.*, 2005 ▶); program(s) used to refine structure: *SHELXL97* (Sheldrick, 2008 ▶); molecular graphics: *ORTEP-3 for Windows* (Farrugia, 1997 ▶); software used to prepare material for publication: *WinGX* (Farrugia, 1999 ▶).

## Supplementary Material

Crystal structure: contains datablocks I, global. DOI: 10.1107/S160053680902011X/is2418sup1.cif

Structure factors: contains datablocks I. DOI: 10.1107/S160053680902011X/is2418Isup2.hkl

Additional supplementary materials:  crystallographic information; 3D view; checkCIF report

## Figures and Tables

**Table 1:** Hydrogen-bond geometry (Å, °)

*D*—H⋯*A*	*D*—H	H⋯*A*	*D*⋯*A*	*D*—H⋯*A*
O5—H5*O*⋯O1^i^	0.96 (11)	1.83 (11)	2.782 (7)	171 (10)
C5—H5*B*⋯O4^i^	0.97	2.49	3.188 (8)	129
C13—H13⋯O3^ii^	0.93	2.46	3.350 (8)	161

Symmetry codes: (i) 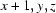; (ii) 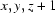.

**Fig. 1.**
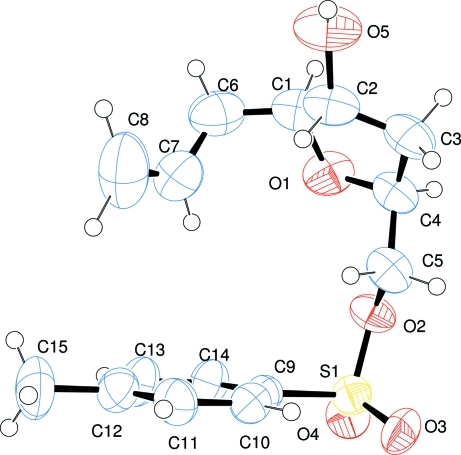
The molecular structure of the title compound, with atom labels and 50% probability displacement ellipsoids for non-H atoms.

**Fig. 2.**
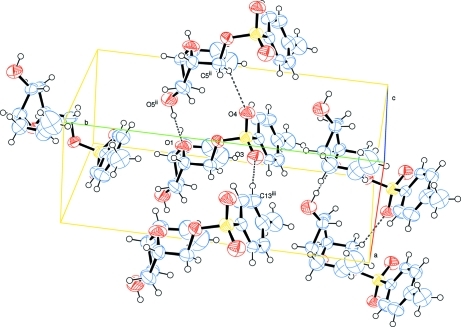
The packing of the title compound, showing molecules connected by O5—H···O1i hydrogen bonds and intermolecular weak interactions (dashed lines).

## supplementary crystallographic information

### Comment

During the course of our investigations led to the synthesis of chiral tetrahydrofurans bearing an allyl group at C1 position (Romero *et al.*, 2006; Sartillo-Melendez *et al.*, 2006; Hernández-Garcia *et al.*, 2009; Paz-Morales *et al.*, 2009), the title compound was obtained and separated by crystallization from the its C1-epimer.

In the crystal structure, the tosyl ring and terminal double bond reach a close distance from each one (C7···C13 and C8···C12 = *ca.* 3.9 Å), with allyl π orbital being perpendicular for those of the aromatic ring. The furane ring (O1/C1–C4) shows an envelope conformation on atom C2 with puckering parameters (Cremer & Pople, 1975) *q*_2_ = 0.348 (8) Å and *φ*_2_ = 72.3 (12)°. The molecules are linked by hydrogen bond [O1···O5 = 2.782 (7) Å] interactions, building a ribbon structure along the [100] direction.

### Experimental

The title compound was obtained from a solution of 1,2-*O*-isopropylidene-α-D-xylofuranose derivative (1.0 mmol) in 10 ml of dry CH_2_Cl_2_. This was treated with allyltrimethylsilane (6.0 mmol) at room temperature over 10 min and the reaction mixture was cooled at 0 °C, then BF_3_OEt_2_ (6.0 mmol) was added dropwise. The reaction mixture was warmed at room temperature over 6 hrs and was treated with saturated aqueous solution of NaHCO_3_ (10 ml). The aqueous layer was extracted three times with CH_2_Cl_2_ (20 ml). The organic phase was dried with MgSO_4_, concentrated and purified by flash chromatography on silica gel (hexane: ethyl acetate). The absolute configuration was established by the structure determination of 1,2-*O*-isopropylidene-α-D-xylofuranose of known absolute configuration of starting material.

### Refinement

The H atom bonded to O atom was located in a difference map and the positional parameters were refined, with *U*_iso_(H) = 1.5*U*_eq_(O). C-bound H atoms were placed in geometrical idealized positions (C—H = 0.93–0.98 Å) and were refined as riding, with *U*_iso_(H) = 1.2*U*_eq_(C) or 1.5*U*_eq_(methyl C).

### Crystal data

**Table:**

C_15_H_20_O_5_S	*F*(000) = 332
*M**_r_* = 312.37	*D*_x_ = 1.275 Mg m^−^^3^
Monoclinic, *P*2_1_	Mo *K*α radiation, λ = 0.71073 Å
Hall symbol: P 2yb	Cell parameters from 40 reflections
*a* = 5.9420 (12) Å	θ = 4.8–24.7°
*b* = 16.966 (3) Å	µ = 0.22 mm^−^^1^
*c* = 8.1980 (19) Å	*T* = 293 K
β = 100.09 (2)°	Prism, colorless
*V* = 813.7 (3) Å^3^	0.6 × 0.4 × 0.3 mm
*Z* = 2	

### Data collection

**Table:**

Bruker P4 diffractometer	*R*_int_ = 0.030
Radiation source: fine-focus sealed tube	θ_max_ = 25.5°, θ_min_ = 2.4°
graphite	*h* = −1→7
2θ/ω scans	*k* = −1→20
2127 measured reflections	*l* = −9→9
1575 independent reflections	3 standard reflections every 97 reflections
1099 reflections with *I* > 2σ(*I*)	intensity decay: 7%

### Refinement

**Table:**

Refinement on *F*^2^	Secondary atom site location: difference Fourier map
Least-squares matrix: full	Hydrogen site location: inferred from neighbouring sites
*R*[*F*^2^ > 2σ(*F*^2^)] = 0.055	H atoms treated by a mixture of independent and constrained refinement
*wR*(*F*^2^) = 0.126	*w* = 1/[σ^2^(*F*_o_^2^) + (0.0261*P*)^2^ + 0.5527*P*] where *P* = (*F*_o_^2^ + 2*F*_c_^2^)/3
*S* = 1.07	(Δ/σ)_max_ < 0.001
1575 reflections	Δρ_max_ = 0.26 e Å^−^^3^
194 parameters	Δρ_min_ = −0.23 e Å^−^^3^
1 restraint	Absolute structure: Flack (1983), 97 Friedel pairs
Primary atom site location: structure-invariant direct methods	Flack parameter: −0.1 (2)

### Special details

**Table:**

Geometry. All s.u.'s (except the s.u. in the dihedral angle between two l.s. planes) are estimated using the full covariance matrix. The cell s.u.'s are taken into account individually in the estimation of s.u.'s in distances, angles and torsion angles; correlations between s.u.'s in cell parameters are only used when they are defined by crystal symmetry. An approximate (isotropic) treatment of cell s.u.'s is used for estimating s.u.'s involving l.s. planes.
Refinement. Refinement of *F*^2^ against ALL reflections. The weighted *R*-factor *wR* and goodness of fit *S* are based on *F*^2^, conventional *R*-factors *R* are based on *F*, with *F* set to zero for negative *F*^2^. The threshold expression of *F*^2^ > 2σ(*F*^2^) is used only for calculating *R*-factors(gt) *etc*. and is not relevant to the choice of reflections for refinement. *R*-factors based on *F*^2^ are statistically about twice as large as those based on *F*, and *R*- factors based on ALL data will be even larger.

### Fractional atomic coordinates and isotropic or equivalent isotropic displacement parameters (Å^2^)

**Table:**

	*x*	*y*	*z*	*U*_iso_*/*U*_eq_	
S1	0.2153 (3)	0.44181 (11)	0.35980 (19)	0.0535 (4)	
O4	−0.0229 (6)	0.4463 (4)	0.3656 (5)	0.0664 (12)	
O2	0.2892 (7)	0.5318 (3)	0.3599 (6)	0.0586 (12)	
C9	0.3645 (10)	0.4054 (4)	0.5478 (7)	0.0456 (14)	
O1	0.5274 (7)	0.6525 (3)	0.5384 (7)	0.0707 (15)	
O3	0.2936 (8)	0.4018 (3)	0.2256 (5)	0.0673 (14)	
O5	1.1076 (9)	0.7133 (4)	0.5830 (9)	0.092 (2)	
C10	0.5693 (10)	0.3653 (4)	0.5528 (8)	0.0488 (15)	
H10	0.6287	0.3575	0.4564	0.059*	
C4	0.5632 (11)	0.6353 (4)	0.3722 (9)	0.0615 (19)	
H4	0.4558	0.6660	0.2926	0.074*	
C11	0.6847 (12)	0.3367 (4)	0.7038 (9)	0.0611 (19)	
H11	0.8222	0.3100	0.7077	0.073*	
C14	0.2741 (12)	0.4168 (4)	0.6894 (8)	0.0614 (19)	
H14	0.1377	0.4441	0.6860	0.074*	
C2	0.9228 (11)	0.6608 (4)	0.5423 (10)	0.065 (2)	
H2	0.9719	0.6073	0.5770	0.077*	
C5	0.5240 (11)	0.5496 (4)	0.3390 (9)	0.0626 (19)	
H5A	0.5446	0.5370	0.2271	0.075*	
H5B	0.6317	0.5186	0.4158	0.075*	
C3	0.8076 (12)	0.6601 (5)	0.3629 (10)	0.075 (2)	
H3A	0.8799	0.6225	0.2994	0.090*	
H3B	0.8110	0.7119	0.3135	0.090*	
C13	0.3905 (13)	0.3868 (5)	0.8364 (9)	0.070 (2)	
H13	0.3274	0.3931	0.9317	0.084*	
C12	0.5976 (12)	0.3476 (5)	0.8486 (8)	0.065 (2)	
C1	0.7298 (11)	0.6868 (5)	0.6323 (10)	0.068 (2)	
H1	0.7159	0.7442	0.6244	0.082*	
C15	0.7242 (14)	0.3163 (6)	1.0118 (9)	0.092 (3)	
H15A	0.7423	0.2603	1.0040	0.138*	
H15B	0.6388	0.3280	1.0980	0.138*	
H15C	0.8719	0.3408	1.0370	0.138*	
C7	0.765 (2)	0.5786 (7)	0.8486 (12)	0.100 (3)	
H7	0.6437	0.5466	0.8007	0.120*	
C6	0.7504 (16)	0.6637 (5)	0.8113 (11)	0.088 (3)	
H6A	0.8857	0.6888	0.8728	0.106*	
H6B	0.6196	0.6849	0.8526	0.106*	
C8	0.938 (2)	0.5455 (8)	0.9454 (12)	0.135 (5)	
H8A	1.0609	0.5760	0.9949	0.162*	
H8B	0.9375	0.4915	0.9645	0.162*	
H5O	1.26 (2)	0.695 (7)	0.578 (14)	0.162*	

### Atomic displacement parameters (Å^2^)

**Table:**

	*U*^11^	*U*^22^	*U*^33^	*U*^12^	*U*^13^	*U*^23^
S1	0.0460 (8)	0.0574 (9)	0.0559 (9)	−0.0012 (9)	0.0053 (7)	0.0015 (10)
O4	0.038 (2)	0.080 (3)	0.081 (3)	−0.001 (3)	0.010 (2)	0.008 (4)
O2	0.044 (2)	0.050 (3)	0.084 (3)	−0.003 (2)	0.017 (2)	0.008 (2)
C9	0.046 (3)	0.043 (3)	0.048 (3)	−0.006 (3)	0.008 (3)	0.002 (3)
O1	0.035 (2)	0.087 (4)	0.093 (4)	−0.009 (3)	0.020 (2)	−0.010 (3)
O3	0.078 (3)	0.079 (3)	0.046 (2)	−0.003 (3)	0.013 (2)	−0.015 (2)
O5	0.050 (3)	0.082 (4)	0.146 (5)	−0.010 (3)	0.022 (4)	−0.015 (4)
C10	0.042 (4)	0.052 (4)	0.054 (4)	−0.004 (3)	0.013 (3)	−0.005 (3)
C4	0.049 (4)	0.052 (4)	0.082 (5)	0.005 (3)	0.009 (4)	0.016 (4)
C11	0.053 (4)	0.059 (5)	0.068 (4)	0.008 (4)	0.000 (3)	0.009 (4)
C14	0.062 (4)	0.073 (5)	0.051 (4)	0.010 (4)	0.013 (3)	−0.006 (3)
C2	0.033 (3)	0.051 (4)	0.109 (6)	−0.001 (3)	0.012 (4)	0.001 (4)
C5	0.048 (4)	0.061 (4)	0.083 (5)	0.004 (4)	0.021 (4)	0.012 (4)
C3	0.056 (4)	0.072 (5)	0.102 (6)	−0.012 (4)	0.028 (4)	0.018 (5)
C13	0.068 (5)	0.089 (6)	0.055 (4)	0.007 (4)	0.017 (4)	−0.007 (4)
C12	0.062 (5)	0.072 (5)	0.057 (4)	−0.013 (4)	0.000 (3)	−0.001 (4)
C1	0.041 (4)	0.061 (5)	0.104 (6)	0.007 (3)	0.017 (4)	0.003 (4)
C15	0.091 (6)	0.109 (7)	0.066 (5)	−0.001 (6)	−0.010 (4)	0.028 (5)
C7	0.117 (9)	0.100 (8)	0.080 (6)	−0.020 (7)	0.014 (6)	−0.011 (6)
C6	0.076 (6)	0.083 (7)	0.108 (7)	−0.011 (5)	0.022 (5)	−0.017 (6)
C8	0.181 (12)	0.135 (10)	0.088 (7)	0.042 (10)	0.023 (8)	0.016 (7)

### Geometric parameters (Å, °)

**Table:**

S1—O4	1.427 (4)	C2—C1	1.533 (9)
S1—O3	1.437 (5)	C2—H2	0.9800
S1—O2	1.589 (5)	C5—H5A	0.9700
S1—C9	1.751 (6)	C5—H5B	0.9700
O2—C5	1.467 (7)	C3—H3A	0.9700
C9—C14	1.375 (8)	C3—H3B	0.9700
C9—C10	1.389 (8)	C13—C12	1.386 (10)
O1—C1	1.433 (8)	C13—H13	0.9300
O1—C4	1.445 (8)	C12—C15	1.512 (9)
O5—C2	1.408 (8)	C1—C6	1.503 (11)
O5—H5O	0.96 (11)	C1—H1	0.9800
C10—C11	1.392 (9)	C15—H15A	0.9600
C10—H10	0.9300	C15—H15B	0.9600
C4—C5	1.490 (10)	C15—H15C	0.9600
C4—C3	1.526 (9)	C7—C8	1.311 (14)
C4—H4	0.9800	C7—C6	1.475 (13)
C11—C12	1.388 (9)	C7—H7	0.9300
C11—H11	0.9300	C6—H6A	0.9700
C14—C13	1.378 (9)	C6—H6B	0.9700
C14—H14	0.9300	C8—H8A	0.9300
C2—C3	1.509 (10)	C8—H8B	0.9300
			
O4—S1—O3	120.5 (3)	H5A—C5—H5B	108.5
O4—S1—O2	103.0 (3)	C2—C3—C4	103.1 (6)
O3—S1—O2	109.1 (3)	C2—C3—H3A	111.1
O4—S1—C9	110.0 (3)	C4—C3—H3A	111.1
O3—S1—C9	109.0 (3)	C2—C3—H3B	111.1
O2—S1—C9	104.0 (3)	C4—C3—H3B	111.1
C5—O2—S1	117.7 (4)	H3A—C3—H3B	109.1
C14—C9—C10	120.9 (6)	C14—C13—C12	122.9 (7)
C14—C9—S1	118.7 (5)	C14—C13—H13	118.6
C10—C9—S1	120.3 (5)	C12—C13—H13	118.6
C1—O1—C4	109.9 (5)	C13—C12—C11	117.5 (6)
C2—O5—H5O	119 (7)	C13—C12—C15	122.0 (7)
C9—C10—C11	119.3 (6)	C11—C12—C15	120.5 (7)
C9—C10—H10	120.4	O1—C1—C6	109.6 (6)
C11—C10—H10	120.4	O1—C1—C2	104.6 (6)
O1—C4—C5	109.0 (6)	C6—C1—C2	117.1 (7)
O1—C4—C3	106.9 (6)	O1—C1—H1	108.4
C5—C4—C3	112.3 (6)	C6—C1—H1	108.4
O1—C4—H4	109.6	C2—C1—H1	108.4
C5—C4—H4	109.6	C12—C15—H15A	109.5
C3—C4—H4	109.6	C12—C15—H15B	109.5
C12—C11—C10	121.0 (7)	H15A—C15—H15B	109.5
C12—C11—H11	119.5	C12—C15—H15C	109.5
C10—C11—H11	119.5	H15A—C15—H15C	109.5
C9—C14—C13	118.4 (6)	H15B—C15—H15C	109.5
C9—C14—H14	120.8	C8—C7—C6	123.7 (12)
C13—C14—H14	120.8	C8—C7—H7	118.1
O5—C2—C3	116.0 (7)	C6—C7—H7	118.1
O5—C2—C1	108.8 (6)	C7—C6—C1	116.7 (8)
C3—C2—C1	102.8 (6)	C7—C6—H6A	108.1
O5—C2—H2	109.7	C1—C6—H6A	108.1
C3—C2—H2	109.7	C7—C6—H6B	108.1
C1—C2—H2	109.7	C1—C6—H6B	108.1
O2—C5—C4	107.5 (5)	H6A—C6—H6B	107.3
O2—C5—H5A	110.2	C7—C8—H8A	120.0
C4—C5—H5A	110.2	C7—C8—H8B	120.0
O2—C5—H5B	110.2	H8A—C8—H8B	120.0
C4—C5—H5B	110.2		
			
O4—S1—O2—C5	−174.5 (5)	O5—C2—C3—C4	−151.9 (6)
O3—S1—O2—C5	−45.4 (5)	C1—C2—C3—C4	−33.3 (8)
C9—S1—O2—C5	70.8 (5)	O1—C4—C3—C2	21.1 (8)
O4—S1—C9—C14	−26.7 (6)	C5—C4—C3—C2	−98.3 (8)
O3—S1—C9—C14	−160.7 (5)	C9—C14—C13—C12	1.9 (12)
O2—S1—C9—C14	83.0 (6)	C14—C13—C12—C11	−2.0 (12)
O4—S1—C9—C10	152.9 (5)	C14—C13—C12—C15	179.0 (8)
O3—S1—C9—C10	18.9 (6)	C10—C11—C12—C13	0.9 (11)
O2—S1—C9—C10	−97.4 (5)	C10—C11—C12—C15	179.9 (7)
C14—C9—C10—C11	−0.4 (9)	C4—O1—C1—C6	−148.3 (6)
S1—C9—C10—C11	−179.9 (5)	C4—O1—C1—C2	−21.9 (8)
C1—O1—C4—C5	122.2 (6)	O5—C2—C1—O1	158.0 (6)
C1—O1—C4—C3	0.6 (8)	C3—C2—C1—O1	34.5 (8)
C9—C10—C11—C12	0.2 (10)	O5—C2—C1—C6	−80.5 (9)
C10—C9—C14—C13	−0.6 (10)	C3—C2—C1—C6	156.1 (7)
S1—C9—C14—C13	178.9 (6)	C8—C7—C6—C1	120.2 (11)
S1—O2—C5—C4	−170.4 (5)	O1—C1—C6—C7	59.6 (11)
O1—C4—C5—O2	58.8 (7)	C2—C1—C6—C7	−59.3 (12)
C3—C4—C5—O2	177.0 (5)		

### Hydrogen-bond geometry (Å, °)

**Table:**

*D*—H···*A*	*D*—H	H···*A*	*D*···*A*	*D*—H···*A*
O5—H5O···O1^i^	0.96 (11)	1.83 (11)	2.782 (7)	171 (10)
C5—H5B···O4^i^	0.97	2.49	3.188 (8)	129
C13—H13···O3^ii^	0.93	2.46	3.350 (8)	161

Symmetry codes: (i) *x*+1, *y*, *z*; (ii) *x*, *y*, *z*+1.
